# Non-classical Congenital Adrenal Hyperplasia Presenting With Severe Androgenic Alopecia: A Case Report

**DOI:** 10.7759/cureus.79012

**Published:** 2025-02-14

**Authors:** Khalid Al Hawsawi, Hamazah Qul, Ameera A Alkhamesi, Sarah M Fageeh

**Affiliations:** 1 Dermatology, King Abdulaziz Hospital, Makkah, SAU; 2 Internal Medicine, King Abdulaziz Hospital, Makkah, SAU; 3 Medicine, King Abdulaziz University, Jeddah, SAU

**Keywords:** acne, alopecia, dhea-s, hirsutism, infertility, nccah

## Abstract

Congenital adrenal hyperplasia (CAH) is an autosomal recessive disorder with cortisol synthesis impairment, commonly due to CYP21A2 gene mutations. Non-classical CAH (NCCAH) presents with hyperandrogenic symptoms such as acne, hirsutism, severe androgenic alopecia, and infertility. We report a 37-year-old female who presented with severe acne, progressive hair loss, and primary infertility despite regular menstrual cycles. Laboratory tests were normal except for elevated 17α-hydroxyprogesterone (17-OHP). Prednisolone was initiated to manage symptoms and address fertility.

## Introduction

Congenital adrenal hyperplasia (CAH) is a spectrum of autosomal recessive conditions marked by different degrees of cortisol impairment, ranging from mild to severe forms caused by a deficiency in one of the five adrenal steroidogenic enzymes needed for cortisol synthesis [1,2]. Disorders involve an imbalance in sex steroid production, either insufficient or excessive, which affects the development of primary or secondary sex characteristics in infants, children, or adults. The most common form of CAH is linked to steroid 21-hydroxylase deficiency, caused by a gene called CYP21A2 [3,4], affecting about 90% of all cases. The clinical presentation is a consequence of deficiency of 21-hydroxylation of progesterone and 17α-hydroxyprogesterone (17-OHP), resulting in a defect in the production of both cortisol and aldosterone so that the pathway is diverted toward the overproduction of androgens [5,6].

We present a case of a 37-year-old female who presented to our dermatology OPD, who happens to be a known case of acne for several years and has had hair loss for four years.

## Case presentation

A 37-year-old Saudi female patient is suffering from primary infertility and severe acne that has been persistent for the last several years. She presented to the dermatology clinic due to significant hair loss that had been ongoing for the past seven years. The hair loss was described as gradual and continuous, without associated symptoms such as itching or pain. There was no history of predisposing causes, such as recurrent high fever, surgery, or major life stressors. The patient denied a family history of hair loss or a similar condition. She was married eight years ago and has not conceived to date. She was investigated for primary infertility, and the cause was not found. She received oral clomiphene citrate treatment. She has no history of thyroid disorders, autoimmune diseases, or anemia. Additionally, she was not on any medications apart from the doxycycline tab and topical anti-acne treatment.

**Figure 1:**
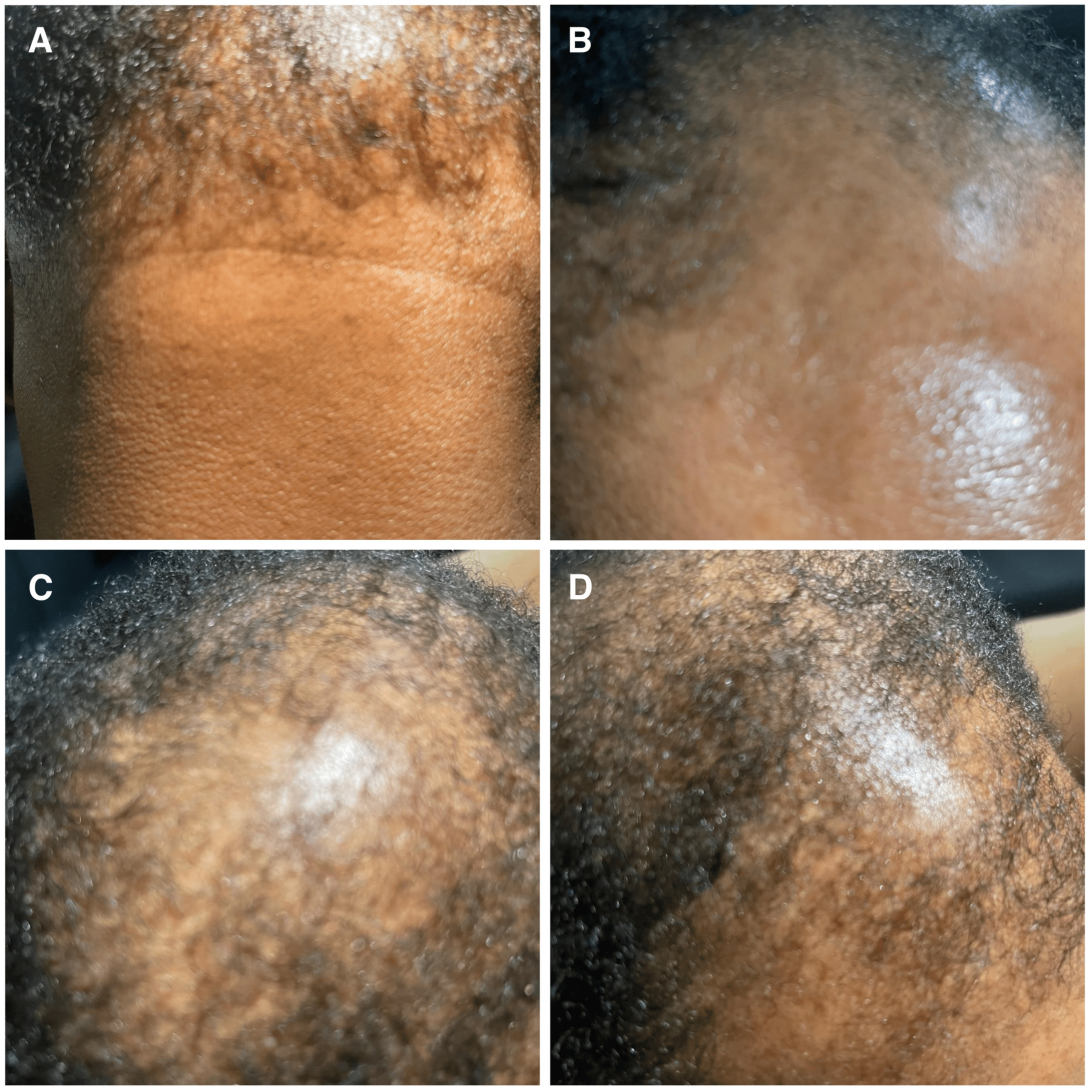
(A) Thinning of the posterior hairline. (B) Forehead with patchy hair loss and smooth, shiny scalp. (C, D) The crown of the head showing significant hair thinning with central bald areas

**Figure 2:**
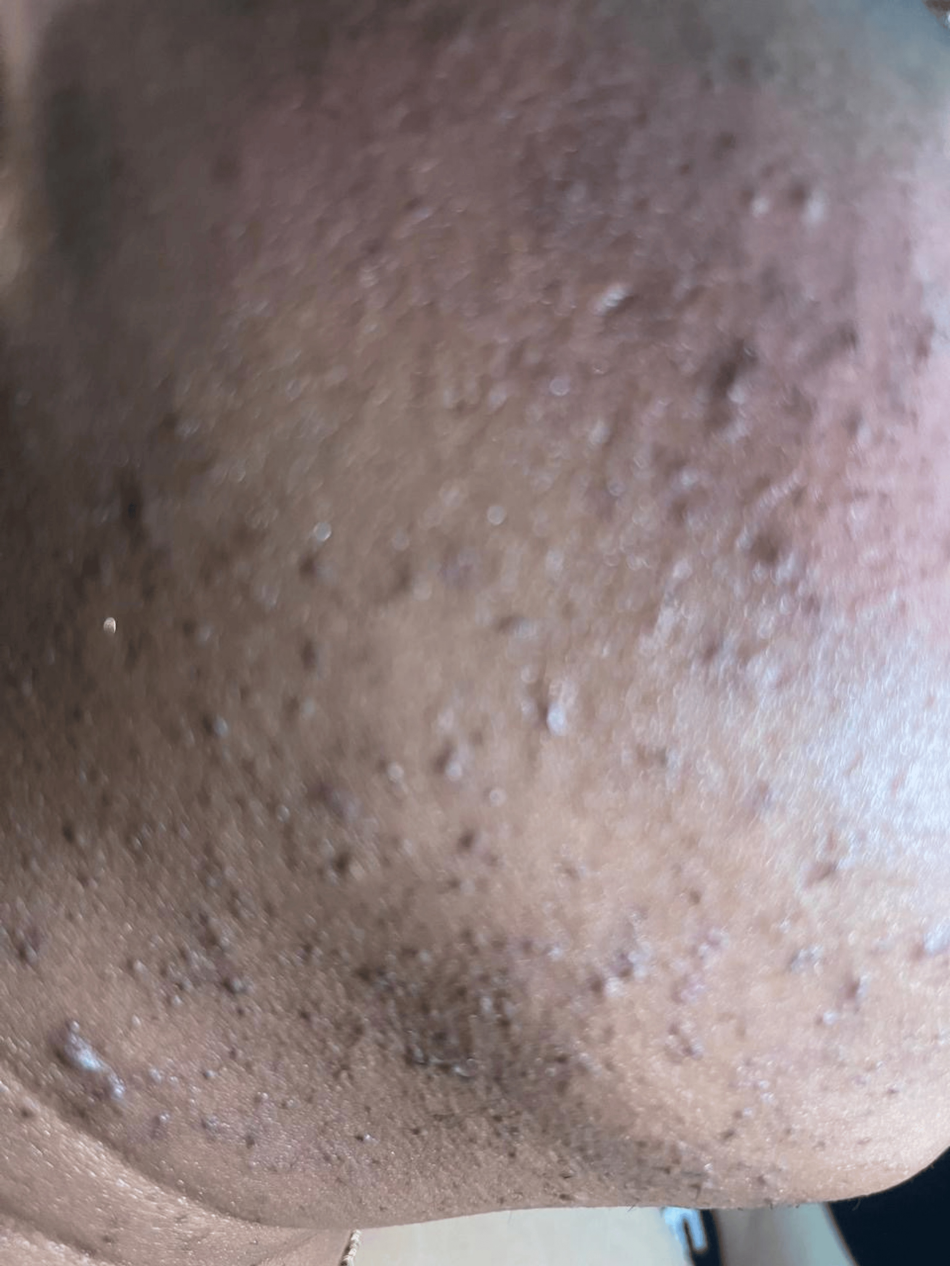
Multiple small papules, hyperpigmented macules, and follicular prominence suggestive of post-inflammatory hyperpigmentation

A review of systems revealed regular menstrual cycles. She denied a history of ambiguous genitalia, symptoms of adrenal insufficiency, or masculinization. There were no changes in her voice or other signs of virilization. Physical examination revealed severe hair loss that is consistent with a severe male pattern of androgenic alopecia (Figure 1). The patient also exhibited severe acne on her face (Figure 2). There were no signs of masculinization, clitoromegaly, or acanthosis nigricans.

Laboratory tests revealed normal white blood cell count, hemoglobin, platelet count, and coagulation profile. The 17-OHP levels were very high. Free testosterone, dehydroepiandrosterone sulfate (DHEA-S), sex hormone-binding globulin (SHBG), and prolactin levels were normal (Table 1).

**Table 1:** Laboratory results

Parameter	Observed value	Reference range
Sex hormone-binding globulin	36.920 nmol/L	25.0–135 nmol/L
Dehydroepiandrosterone sulfate	113.2 µg/dL	60.9–337 µg/dL
Testosterone	1.7 pg/mL	0.0–4.20 pg/mL
17α-hydroxyprogesterone (chemiluminescent immunoassay)	8.6 ng/mL	Follicular phase: 0.1–0.8 ng/mL
Prolactin	7.574 ng/mL	3.8–30.7 ng/mL

The clinical manifestations and laboratory results were consistent with the patient's non-classical CAH (NCCAH) diagnosis. The patient was started on prednisolone 5 mg/day for three months to inhibit adrenocorticotropic hormone (ACTH) and to control symptoms of hyperandrogenism. The patient was put on periodic follow-ups. The patient was seen post-treatment in a follow-up visit, showed improvement, and is still being followed. She has scheduled follow-up visits.

## Discussion

Many individuals with NCCAH remain asymptomatic through childhood and adolescence. They are often diagnosed with CAH incidentally following the identification of a family member with this condition. Women with NCCAH seek medical attention when they start to develop the symptoms of androgen excess manifestations like acne, alopecia, and hirsutism. 17-OHP serum level is a primary marker for NCCAH [7,8].

**Figure 3:**
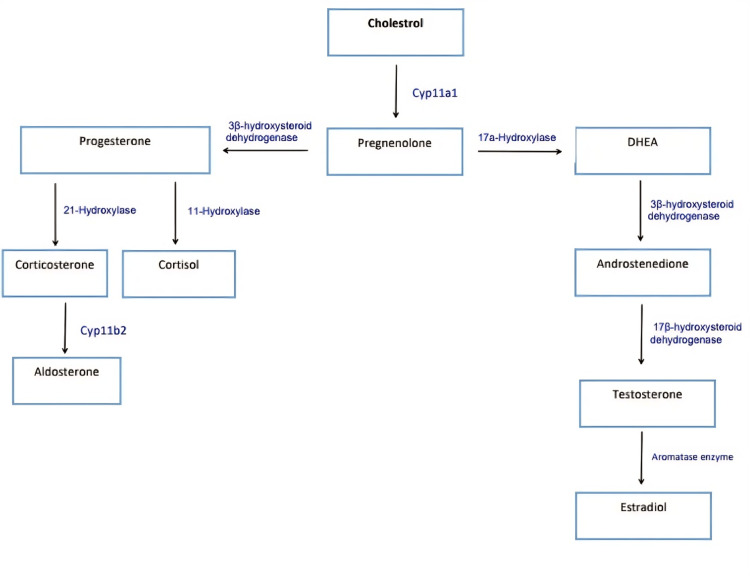
Steroidogenesis pathway DHEA: dehydroepiandrosterone

In CAH, the genetic defect in one of the enzymes responsible for synthesizing corticosteroids and/or mineralocorticosteroids leads to low blood levels of corticosteroids and/or mineralocorticosteroids. This will divert the pathway toward excessive production of androgens, the steroidogenesis pathway [9], described in Figure 3.

This will also stimulate the pituitary gland to secrete ACTH to stimulate the adrenal glands to secrete corticosteroids and/or mineralocorticosteroids. However, the adrenal glands are unable to secrete corticosteroids and/or mineralocorticosteroids, so the results will be the persistence of the low corticosteroids and/or mineralocorticosteroids, high levels of androgens, and very high serum level of ACTH. The patient did not undergo the following tests: blood level of ACTH, early morning cortisol serum level, or ACTH stimulation test. However, the very high level of 17-OHP (8.6 ng/ml), normal reference (0.1-0.8 ng/ml), and the clinical picture were used to diagnose NCCAH.

This decision to start treating this patient was based on several factors, including severe hair fall, severe acne, and the patient’s desire for pregnancy and her long-standing history of infertility, despite multiple attempts at ovarian stimulation using clomiphene citrate. Prednisolone was chosen due to its low-risk profile and its ability to inhibit ACTH and manage the symptoms of hyperandrogenism effectively.

Emini et al. described a woman (27 years old) with a two-year history of severe female pattern hair loss, hirsutism, and weight loss but no menstrual irregularities. High 17-OHP and androgen levels confirmed NCCAH in that patient [10]. In contrast, our patient exhibited severe acne, severe male pattern androgenic alopecia, and primary infertility, with regular menstrual irregularities alongside normal androgen levels, which made the diagnosis challenging, reinforcing the value of 17-OHP testing in hyperandrogenic conditions.

Another case reported by Livadas and Bothou described NCCAH with hirsutism and menstrual irregularities, with elevated 17-OHP post-ACTH stimulation confirming the diagnosis of CAH [11]. However, unlike that case, our patient had regular menstrual cycles and had no hirsutism.

Monni et al. described an 11-year-old girl who presented with genital ambiguity, excessive growth of hair (hirsutism) on the pubis, axilla, face, and lower limbs, as well as clitoromegaly, deepening of voice, and excessive sweating over four years. Her hormonal profile showed an elevation in 17-OHP, confirming the diagnosis of NCCAH. The patient also had a marginally elevated serum testosterone and DHEA-S, consistent with hyperandrogenism [12].

In contrast, our patient, a 37-year-old woman, had a history of primary infertility and severe acne but presented with male-pattern androgenic alopecia (Figure 1) and severe acne on her face (Figure 2). While her 17-OHP was significantly elevated, her free testosterone, DHEA-S, SHBG, and prolactin levels were normal, which made the diagnosis more complex. Unlike the 11-year-old girl, our patient did not show signs of clitoromegaly, deepened voice, or hirsutism, which are more typical of classic NCCAH or early-onset forms.

In terms of diagnostic challenges, the normal androgen levels in our patient, despite elevated 17-OHP, made her case more complex to diagnose, particularly because NCCAH is often associated with elevated testosterone or other androgen markers. In contrast, the 11-year-old girl presented with clear signs of hyperandrogenism, which made the diagnosis more straightforward, with 17-OHP levels confirmed by ACTH stimulation tests.

Our case is unique due to unusual clinical presentation (regular menstrual cycles and no hirsutism) and the diagnostic difficulty posed by normal serum androgens. This highlights the importance of detailed hormonal evaluation, specifically 17-OHP serum level, in patients with clinical presentation of hyperandrogenemia.

## Conclusions

This case highlights the importance of considering NCCAH in patients presenting with hyperandrogenic symptoms such as severe acne, androgenic alopecia, hirsutism, and unexplained infertility, even in the presence of normal routine hormonal profiles. The elevated 17-OHP levels were a key diagnostic marker, underscoring the need for comprehensive endocrinological assessment in challenging presentations. Early diagnosis and targeted management, including glucocorticoid therapy, can significantly improve patient outcomes and address associated infertility concerns.
